# Transcervical Dumon Stenting via Longitudinal Tracheotomy for Recurrent Upper Tracheal Restenosis after Failed Resection

**DOI:** 10.5761/atcs.cr.26-00057

**Published:** 2026-05-08

**Authors:** Mu-Chou Lin, Ying-Yuan Chen

**Affiliations:** Division of Thoracic Surgery, Department of Surgery, National Cheng Kung University Hospital, College of Medicine, National Cheng Kung University, Tainan, Taiwan

**Keywords:** endobronchial tuberculosis, longitudinal tracheotomy, silicone stent, tracheal resection, upper tracheal stenosis

## Abstract

Post-tuberculosis airway stenosis can cause fixed cicatricial narrowing and long-term disability. We report a female patient with recurrent upper tracheal restenosis despite 2 prior resections, who presented decades later with respiratory failure; intubation was impossible, necessitating emergency tracheostomy. Imaging and bronchoscopy revealed pinhole stenosis near the second tracheal ring and left main bronchial stenosis with a destroyed left lung. The patient remained tracheostomy-dependent and aphonic after infection control. Because repeat resection was high-risk and endoscopic stent delivery was infeasible, a Dumon silicone stent was inserted via transcervical longitudinal tracheotomy under direct vision. Airway patency and phonation improved, and follow-up showed a stable stent without major complications. This open approach may serve as a salvage surgery when endoscopic delivery is impossible.

## Introduction

Endobronchial tuberculosis can progress from mucosal inflammation to fixed cicatricial tracheobronchial stenosis, resulting in chronic dyspnea, stridor, and long-term functional impairment.^1)^ Surgical resection with end-to-end anastomosis is often used for benign tracheal stenosis when technically feasible, and surgical airway plasty has shown acceptable outcomes in selected patients.^2)^ However, some patients develop recurrent stenosis or are unsuitable for further resection, necessitating alternative airway reconstruction strategies.

## Case Report

A 56-year-old woman had long-standing complex airway stenosis secondary to endobronchial tuberculosis. Decades earlier, in 1988, she underwent upper tracheal segmental resection combined with proximal left main bronchial sleeve bronchoplasty at our institution. Early restenosis occurred at both sites, and a repeat upper tracheal resection was performed 1 month later. Despite reoperations, persistent high-level upper tracheal restenosis and chronic stridor limited her daily activities for nearly 3 decades.

**Fig. 1:**
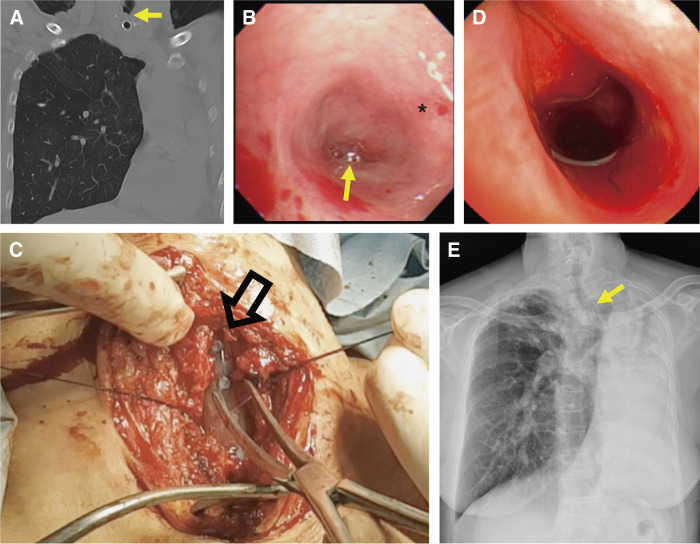
Perioperative imaging and bronchoscopic findings of refractory upper tracheal stenosis managed with open Dumon stenting. (**A**) Preoperative coronal chest computed tomography scan showing severe upper tracheal stenosis (arrow) above a Shiley tracheostomy tube, with a destroyed left lung. (**B**) Preoperative bronchoscopy demonstrating the cricoid cartilage (*) and pinhole upper tracheal stenosis (arrow). (**C**) Intraoperative image showing a longitudinal tracheotomy over the stenotic segment (hollow arrow) with a Dumon silicone stent positioned across the lesion. (**D**) Postoperative bronchoscopy showing the proximal stent margin at the level of the cricoid cartilage with a patent tracheal lumen. (**E**) Chest radiograph at the 6-year follow-up demonstrating the stent in situ (arrow) with maintained position and airway patency.

In 2017, she presented to our emergency department with an acute airway infection complicated by respiratory failure. Endotracheal intubation was impossible because of critical airway narrowing, requiring emergency tracheostomy. After stabilization, computed tomography and flexible bronchoscopy revealed a pinhole-like stenosis around the second tracheal ring (**Figs. 1A** and **1B**), proximal left main bronchial stenosis with a destroyed left lung, and right main bronchial narrowing with a post-pneumonectomy–like configuration. After infection resolution, she remained tracheostomy-dependent and aphonic, with minimal airflow across the vocal cords. Six months later, she found tracheostomy dependence unacceptable and sought decannulation to regain phonation.

Airway reassessment confirmed persistent high-level upper tracheal stenosis. Proximal left main bronchial stenosis was considered clinically irrelevant because the left lung was nonfunctional, and the right main bronchial narrowing was well tolerated without significant symptoms; therefore, intervention was directed solely at the upper tracheal lesion. Given her history of 2 failed tracheal resections, further resection was considered unlikely to succeed and carried substantial risk, including recurrent laryngeal nerve injury and anastomotic complications in a heavily scarred field. Endoscopic intervention was also deemed impractical because the stenosis was extremely tight and located high in the trachea; even balloon dilation was expected to be technically challenging, and rigid bronchoscopic delivery of a silicone stent would likely be unsafe without first achieving a sufficiently patent lumen. Therefore, silicone stent placement via an open cervical approach was planned.

Under general anesthesia, the cervical trachea was exposed via a transverse collar incision. The tracheostomy tube was temporarily exchanged for a 6.0-mm non-kinking endotracheal tube to maintain ventilation during the initial dissection. A longitudinal anterior tracheotomy was extended proximally from the tracheostomy site to traverse the stenotic segment, allowing direct exposure of the narrowed lumen. After intraoperative measurement, a straight 14 × 60-mm Dumon silicone stent (Novatech SA, La Ciotat, France) was prepared for insertion. During deployment, the anesthesiologist advanced an oral endotracheal tube to the tracheotomy field, and the Dumon stent was loaded over the tube. Ventilation was briefly interrupted while the initial tube was removed. The oral tube was then guided into the distal trachea by the operator, ventilation was resumed, and the stent was gradually adjusted to expand the stenosis (**Fig. 1C**). Because distal tracheal intubation and ventilation remained immediately achievable throughout the procedure, extracorporeal support was not considered necessary. Correct stent positioning and luminal patency were confirmed intraoperatively by bronchoscopy (**Fig. 1D**). The tracheal wall was primarily approximated over the stent using interrupted 3-0 monofilament absorbable sutures (Monocryl; Ethicon Inc., Somerville, NJ, USA). The sutures were tied carefully to avoid excessive tension or compression of the stent, and the closure was further reinforced by approximation of the bilateral strap muscles using interrupted 3-0 braided absorbable sutures (Polysorb; Medtronic, Minneapolis, MN, USA). Two Jackson–Pratt drains were placed, and prophylactic antibiotics were administered postoperatively.

The patient was successfully extubated with marked improvement in airway patency and respiratory comfort. Bronchoscopy 5 days postoperatively confirmed a stable stent position without migration. The drains were removed on postoperative day 5 after no output was observed and no signs of surgical site infection were noted.

She was discharged uneventfully and subsequently regained functional daily activities and maintained clinical stability on long-term follow-up. During 7 years of follow-up, the stent remained in situ without migration, granulation, or infection (**Fig. 1E**). She died after an out-of-hospital cardiopulmonary arrest of unclear etiology. Although return of spontaneous circulation was achieved, she remained comatose and died after intensive care. No stent-related airway complication was identified during emergency airway management or the subsequent hospital course.

## Discussion

Post-tuberculosis tracheobronchial disease can progress from endobronchial inflammation to fixed cicatricial stenosis with substantial long-term morbidity.^1)^ Tracheal resection with end-to-end anastomosis remains the standard definitive surgical approach for post-tuberculosis tracheal stenosis. However, in select patients—particularly those with long-segment stenosis or who cannot tolerate resection due to limited physiologic reserve—airway stenting can provide immediate and durable relief of symptoms and may also serve as a temporizing or salvage strategy.^3,4)^

Silicone stents are typically deployed using rigid bronchoscopy but require a lumen that can be safely dilated or ablated to permit endoscopic delivery.^5–7)^ The presented case illustrates a scenario in which the stenosis was so critical and high that even balloon dilation was expected to be difficult, making standard endoscopic delivery impractical. When the lesion is anatomically accessible in the cervical trachea, transcervical longitudinal tracheotomy can provide a direct route for stent insertion.

This open approach offers several pragmatic advantages. Direct exposure provides visualization of the stenotic segment and facilitates precise seating of the Dumon stent across the lesion. Airway management is simplified during stent deployment by maintaining ventilation through an endotracheal tube while the stent is inserted under direct control, with immediate bronchoscopic confirmation. Although extracorporeal membrane oxygenation–supported tracheal reconstruction has been described for severe tracheal stenosis,^8)^ it was not required in our case because ventilation was maintained by stepwise endotracheal tube exchange, with a brief interruption lasting less than 1 min, and the distal trachea remained accessible for rescue re-intubation. These features are particularly relevant when repeated endoscopic manipulation is undesirable or unsafe because of near-total stenosis. Additionally, proximal tracheal stenting requires careful attention to cranial positioning. While the proximal stent margin must traverse the stenotic segment to achieve adequate expansion, the upper edge should ideally be positioned at least 1 cm below the vocal folds, when feasible, to reduce irritation-related granulation and preserve phonatory function.^9)^

Long-term indwelling silicone stenting is feasible despite stent-related events, such as migration, granulation tissue formation, and mucostasis. These complications are usually manageable with structured follow-up and timely bronchoscopic care.^10)^ No postoperative corticosteroids or anti-fibrotic agents were administered in this case. Given the recent tracheal closure and concerns regarding wound healing and infection, we elected not to use postoperative corticosteroids. If recurrent airway compromise occurs, management should be tailored to the underlying cause. This may include bronchoscopic stent repositioning for migration and endoscopic treatment, such as laser ablation or cryotherapy, for granulation tissue. If these measures are not feasible or fail to restore airway patency, long-term airway support with a tracheal T-tube or reinsertion of a tracheostomy tube may be required as salvage options. Fortunately, no granulation tissue formation or restenosis was observed during follow-up. When stent removal is possible, reassessment after stabilization may be appropriate; however, some patients may remain stent-dependent as durable palliation or salvage reconstruction.

## Conclusion

Overall, transcervical stent placement via longitudinal tracheotomy is a practical salvage option for surgically ineligible patients with critically tight cervical tracheal stenosis when endoscopic stent delivery is infeasible.

## Declarations

### Data availability

The data that support the findings of this study are available from the corresponding author upon reasonable request.
